# Explore the Protective Role of Obesity in the Progression of Myocardial Infarction

**DOI:** 10.3389/fcvm.2021.629734

**Published:** 2021-03-25

**Authors:** Siyuan Zhao, Rongyuan Cao, Shuhua Zhang, Yan Kang

**Affiliations:** ^1^Department of Cardiology, Second People's Hospital of Lianyungang, Lianyungang, China; ^2^Human Biochemical Genetics Section, National Institutes of Health, Bethesda, MD, United States

**Keywords:** obesity, myocardial infarction, mega-analysis, functional networks, gene set enrichment analysis

## Abstract

Obesity has been shown as a risk factor to increase the incidence of myocardial infarction (MI). However, obesity has also been linked to the decreased mortality of acute MI with unknown mechanisms. Here, we firstly used large-scale literature data mining to identify obesity downstream targets and MI upstream regulators with polarity, based on which an obesity-MI regulatory network was constructed. Then, a gene set enrichment analysis was conducted to explore the functional profile of the genes involved in the obesity-MI regulatory networks. After that, a mega-analysis using MI RNA expression datasets was conducted to test the expression of obesity-specific genes in MI patients, followed by a shortest-path analysis to explore any potential gene-MI association. Our results suggested that obesity could inhibit 11 MI promoters, including NPPB, NPPA, IRS1, SMAD3, MIR155, ADRB1, AVP, MAPK14, MC3R, ROCK1, and COL3A1, which were mainly involved in blood pressure-related pathways. Our study suggested that obesity could influence MI progression by driving multiple genes associated with blood pressure regulation. Moreover, PTH could be a novel obesity driven gene associated with the pathogenesis of MI, which needs further validation.

## Introduction

The continuous increase in obesity has become a global epidemic, which is attracting more attention than ever (1, 2). Obesity is not only the incentive to induce pathologies of diabetes mellitus, arthritis, hypertension, and certain cancers (1) but also a risk factor for cardiovascular disease, notably myocardial infarction (MI) (2–4). For example, an INTERHEART involving 15,152 cases and 14,820 controls from 52 countries showed that abdominal obesity increased the risk of acute MI in all patients regardless of age, sex, and population regions (4). A prospective cohort study comprising 101,510 participants (age between 18 and 98 years) demonstrated that metabolically healthy obese subjects showed a substantially higher risk of MI incidence in comparison with normal-weight subjects (5). In addition, a meta-analysis involving 36, 803 participants suggested that there was a significant association between overweight and MI (1). However, some clinical studies showed that obese patients with acute MI presented lower mortality than non-obese acute MI patients, which suggested a protective role of obesity in the progression of MI (6, 7). However, so far, the underlying mechanism is largely unknown.

At the genetic level, previous studies showed that multiple genes were associated with both obesity and MI (8–12). For example, the overexpression of TNF is highly prevalent in obesity (9) and plays an important role in the pathogenesis of MI (10). Also, the gene BDNF has been associated with the development of obesity (11), while the deactivation of BDNF could promote cardiac remodeling after MI and improve post-MI survival (12). These previous findings enable the possibility of constructing biology networks to understand the roles of obesity in MI progression.

To address this issue here, we first conducted a literature data mining to identify specific networks connecting obesity and MI. Then, a mega-analysis using multiple MI RNA expression data was conducted to evaluate the genes encoded in the networks. Our results identified multiple biology networks and potential pathways that might add an explanation to the lower mortality of obese acute MI patients.

## Materials and Methods

### Identify Obesity-MI Connection Network

We used the literature-based Elsevier Pathway Studio (www.pathwaystudio.com) knowledge database to identify common genes that were downstream targets of obesity and also up-stream regulators of MI. Each disease-gene relationship has at least three supporting references. A quality control process was enforced to remove unreliable relationships and relationships with unclear polarity. These relation data were then used to compose the literature-based network to explore the possible mechanisms where obesity could play a role in MI progression. To note, in this study, the term “obesity” was defined by Pathway Studio Ontology, which refers to patients with body mass index (BMI) equal to or higher than 30 (www.pathwaystudio.com).

### Selection of MI RNA Expression Datasets for Mega-Analysis

For the obesity down-stream targets that have not been implicated with MI, we conducted a mega-analysis using MI RNA expression datasets, with the purpose to test the expression changes of these genes in the case of MI. The MI RNA expression datasets were collected from the online GEO database (https://www.ncbi.nlm.nih.gov/geo/). The keyword “myocardial infarction” was used on the initial search, and 678 MI related studies with a series dataset were identified. After downloading the original datasets, we outlined the mega data of the identified datasets and selected a sub-set using the following criteria: (1) The dataset was array expression data; (2) The organism of the dataset was Homo sapiens; (3) The study was designed as MI case vs. healthy control; (4) The original data and the corresponding format file were downloadable; (5) sample size was bigger than 10. Seven datasets satisfied the above criteria and were included in the mega-analysis, as shown in Table 1.

**Table 1:** The seven MI expression datasets selected for meta-analysis.

**GEO ID**	**nControl**	**nCase**	**nSample**	**Country**	**Study age (years)**
GSE24591	4	34	38	Italy	3
GSE60993	7	17	24	South Korea	5
GSE66360	50	49	99	USA	5
GSE60993	7	10	17	South Korea	5
GSE34198	48	49	97	Czech Republic	6
GSE48060	21	31	52	USA	6
GSE62646	14	84	98	Poland	6

### Mega-Analysis Models and Reports

In this part, results from both random- and fixed-effect models were used and compared (13). For each gene, the mega-analysis calculated the effect size in terms of gene expression log2 fold-change (LFC). To determine the heterogeneity of the datasets, between- and within-study variances were calculated and compared. When the total variance Q was no-bigger than the expected value of the between-study variances (df), the model sets the ISq (percentage of the within- over between-study variance) to zero. In this case, the fixed-effect model, instead of the random-effects model, will be selected for the mega-analysis. The significant genes from mega-analysis were reported, which satisfy the following criteria: LFC>1 or <–1; *p* < 0.05. All analyses were performed using Matlab (R2017a version).

### Gene Set Enrichment Analysis

To test the functional profile of the genes involved in the obesity-MI regulation network, we conducted a gene set enrichment analysis (GSEA) against the Pathway Studio pathways and Gene Ontology (GO; http://geneontology.org) terms (14). The selection of significantly enriched pathways including (1) the enrichment *p*-value passed the false discovery ratio test (*q* = 0.005); (2) the number of genes involved≤1,000; (3) the overlap percentage≥2%.

## Results

### Biology Network Connecting Obesity and MI

**Figure 1:**
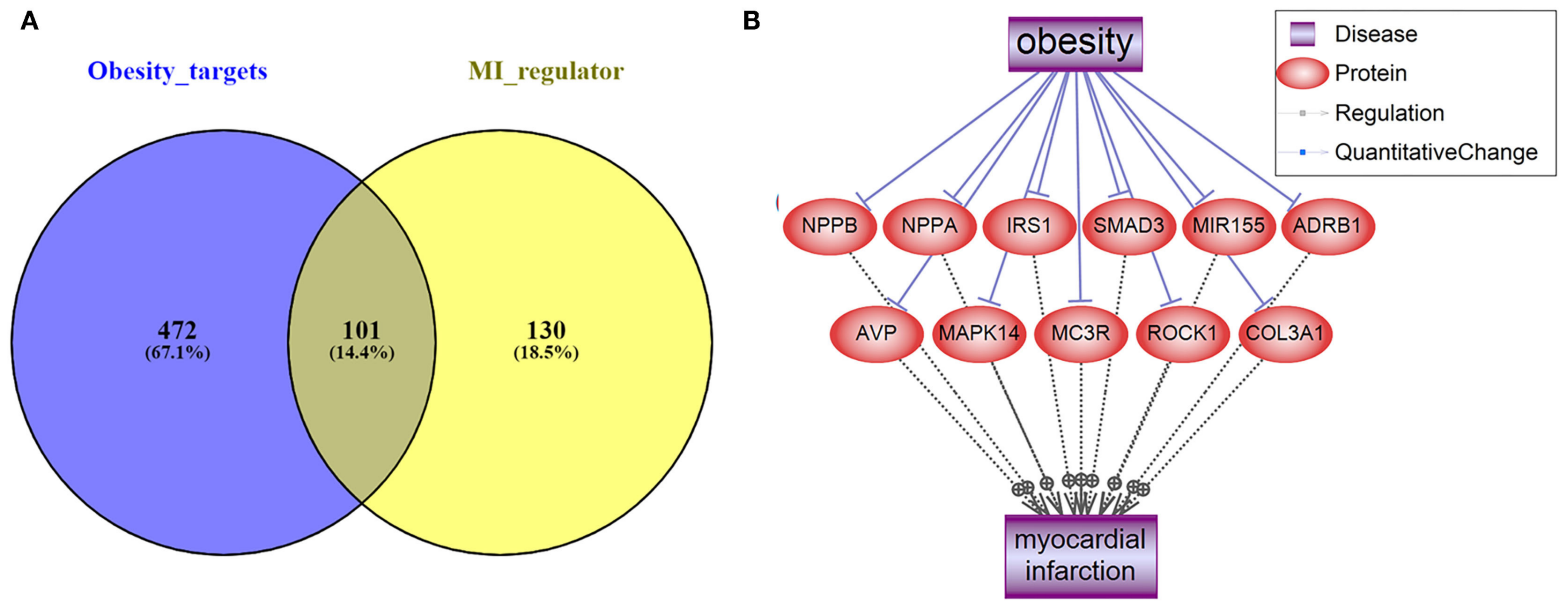
Literature-based data mining results. **(A)** The Venn diagram of obesity targets and myocardial infarction regulators. **(B)** Obesity → MI Inhibitive networks constructed by MI promoters.

As shown in Figure 1A, we used the literature-based knowledge database to identify common genes that were downstream targets of obesity and also up-stream regulators of MI. In total, 573 genes have been identified as obesity targets with polarity, and 231 genes were identified as MI regulators, with 101 shared genes. Each of these relationships was based on at least three supporting references. We presented the details of the gene lists in Supplementary Materials: ObesityTargets and MIRegulators. Out of the 101 genes, we identified 11 MI promoters that were inhibited by obesity, including NPPB, NPPA, IRS1, SMAD3, MIR155, ADRB1, AVP, MAPK14, MC3R, ROCK1, and COL3A1. The genes were employed to construct the Obesity → MI Inhibitive network, as shown in Figure 1B. However, no MI inhibitor was found to be activated by obesity. We provided the detailed information in Supplementary Materials: Obesity_MI_Networks, including the titles and the sentences where these relationships have been identified.

### Functional Enrichment Analysis

**Figure 2:**
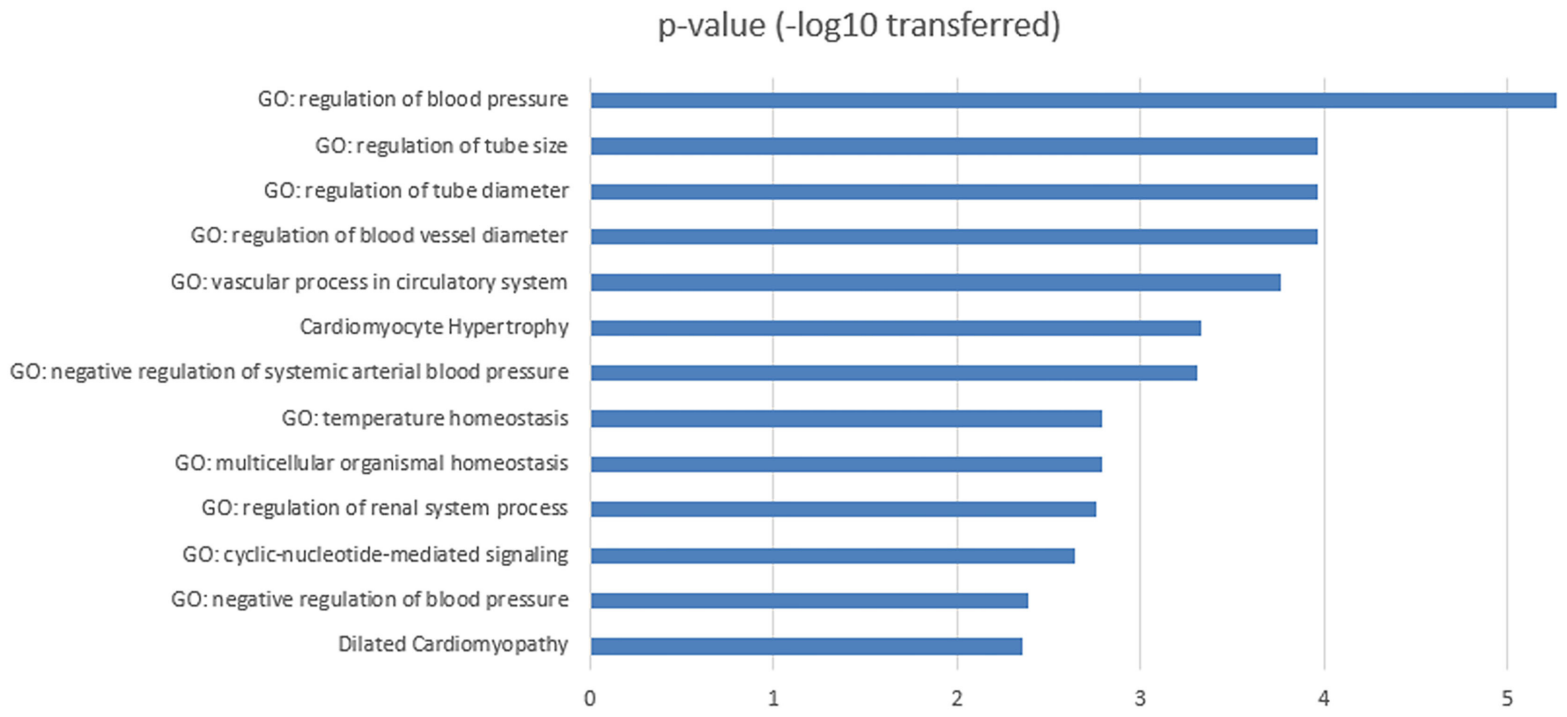
Functional enrichment analysis for the 11 MI-promoters inhibited by obesity within obesity-MI regulatory network.

To investigate the biological functions of the 11 genes (Figure 1B) within the obesity-MI regulatory network, a GSEA was executed by using Pathway Studio. Thirteen GO terms passed the enrichment significance criteria, as shown in Figure 2. We presented the details of these enrichment results in Supplementary Materials: GSEA. These GO terms were mainly related to blood pressure. A previous study showed that acute MI Patients with lower mean blood pressure (<79 mmHg) presented a significantly higher risk of in-hospital mortality (15), while the average systolic and diastolic blood pressure was significantly related to obesity (16). Our results were consistent with these previous findings and partially explained the lower modality rates of acute MI patients with obesity (6, 7).

### Mega-Analysis for MI-Specific Genes

**Figure 3:**
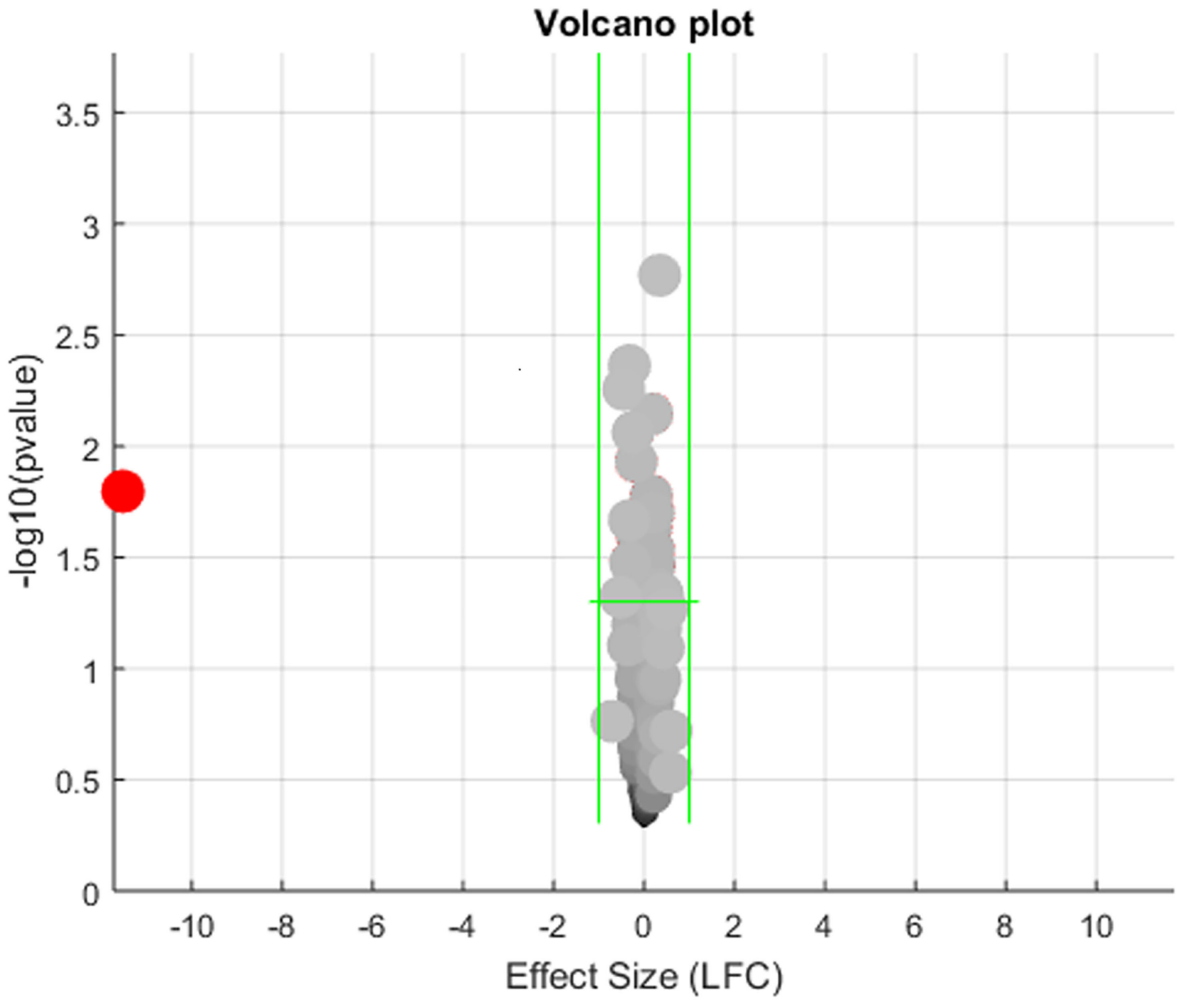
Volcano plot of the mega-analysis results on the 472 obesity regulators but not implicated with myocardial infarction.

The expression levels of the 472 genes that were obesity-targets but not implicated in MI (Figure 1A) were tested in the seven MI RNA expression datasets with a mega-analysis. Results showed that only one gene, PTH, passed the significant gene selection criteria (*p*-value = 0.016 & LFC = −11.52), as shown in Figure 3. We presented the details of the results in Supplementary Materials Mega-analysis.

**Figure 4:**
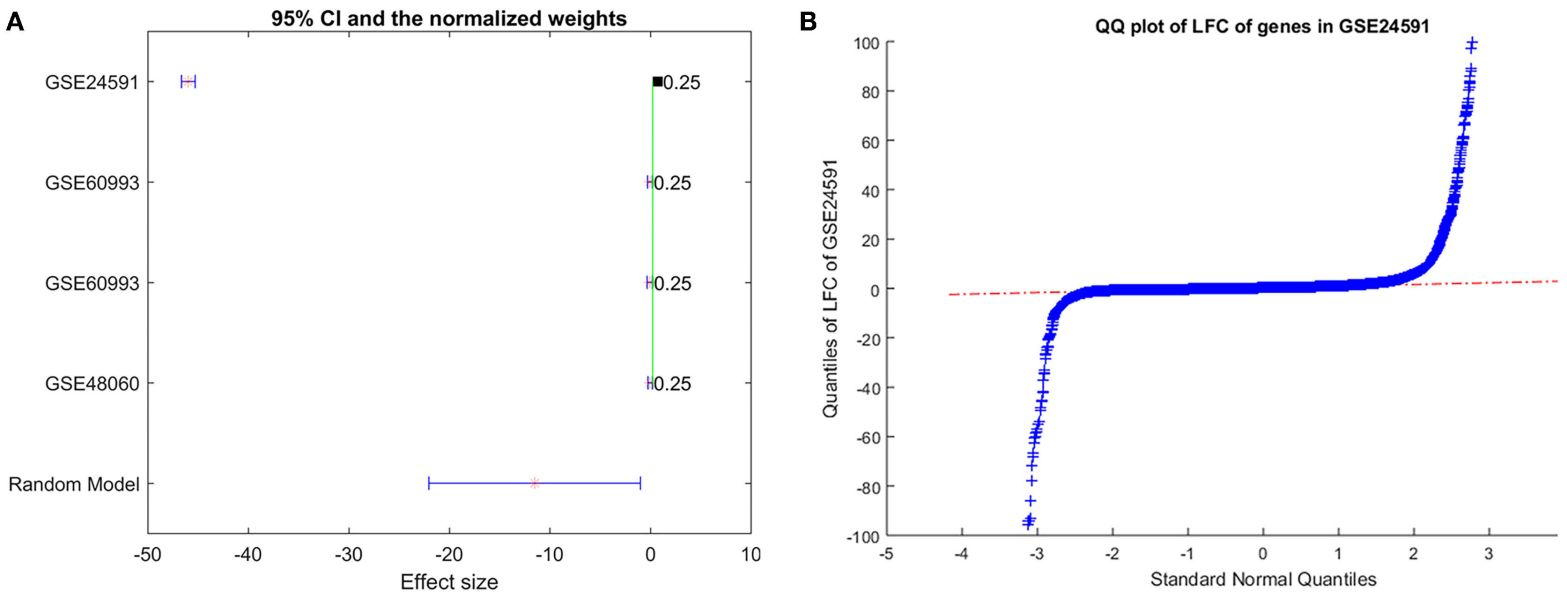
Expression of gene PTH analysis results. **(A)** Forest plot of the mega-analysis results of PTH; **(B)** QQplot of the dataset GSE24591, which includes gene PTH.

However, we noted there was significant between-study variance for the expression of PTH in the case of MI, as shown in Figure 4A. Specifically, the major difference was coming from the dataset GSE24591, which demonstrated a significantly lower expression of PTH (LFC = −45.97; *p*-value<1E-324). To test if the dataset GSE24591 has a systematical bias to generate the significant “outlier”-like expression change of the gene PTH, we conducted a QQplot as shown in Figure 4B. Results showed that the majority of the gene in dataset GSE24591 follow a standard Normal distribution, which suggested that the significantly low PTH expression was not due to a systematical bias.

**Figure 5:**
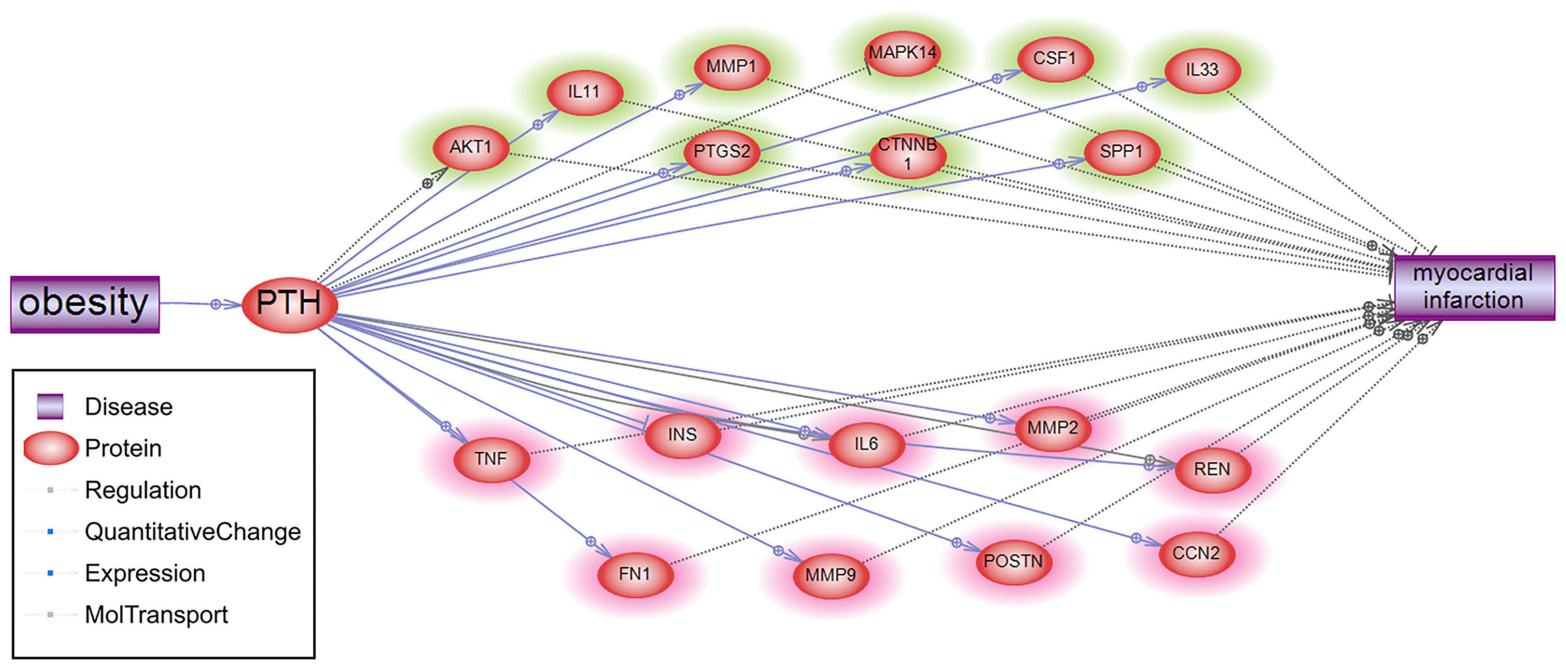
Shortest path connecting obesity target PTH and MI.

To explore the potential role of PTH in the pathological development of MI, we conducted a shortest path analysis that reveals multiple molecules connecting PTH and MI, as shown in Figure 5. Network in Figure 5 also showed that obesity could activate PTH. However, PTH presented mixed roles for MI. On the one hand, PTH could promote multiple MI inhibitors (molecules highlighted in green) to suppress MI development. On the other hand, PTH might also activate several other MI promoters (molecules highlighted in red) that possibly facilitates MI development. Therefore, further study is needed to test the specific role of PTH in MI.

## Discussion

Many previous studies showed that obesity was involved in the pathologies of multiple cardiovascular diseases, including MI (2–4). Meanwhile, some other studies also suggested that obesity was associated with a lower mortality rate of acute MI patients (6, 7). Here, we focused on the exploration of any potential protective role of obesity in MI progression. Network analysis showed that obesity could inhibit 11 MI promoters and activate one potential MI regulator (PTH). The 11 MI promoters' role was supported by multiple previous studies, including NPPB, NPPA, IRS1, SMAD3, MIR155, ADRB1, AVP, MAPK14, MC3R, ROCK1, and COL3A1. In contrast, the PTH-MI relation was surfaced by our mega-analysis that was not implicated before, and our network analysis results could not confirm its role.

The functional network identified in this study supported the protective role of obesity in MI progression. As shown in the obesity → NPPB (Brain natriuretic peptide) or NPPA (Atrial natriuretic peptide) → MI pathway, both natriuretic peptides were belonged to the MI promotors but inhibited by obesity. NPPA/NPPB are members of the natriuretic peptide family that encode atrial natriuretic peptide. The increased secretion of NPPA/NPPB after MI (17, 18) may reflect the degree of left ventricular dysfunction (18), while NPPB levels are decreased in obese individuals (19). Moreover, accumulating evidence showed that ROCK up-regulation plays an important role in the pathogenesis of MI (20), and the inhibition of ROCK suppresses the development of the disease (21). ROCK expression was found to be significantly reduced in obese subjects (22). These findings indicate an obesity → ROCK → MI pathway that supports the suppression role of obesity in MI progression. More references regarding the obesity-driven network were presented in Supplementary Materials: Obesity_MI_Networks.

GSEA results showed that the 11 obesity-driven MI promoters were mostly enriched in the pathways related to blood pressure regulation. The blood pressure is recorded as two numbers: systolic blood pressure (SBP) and diastolic blood pressure (DBP). An inverse relationship between SBP at admission and in-hospital mortality of acute MI patients has been established (15, 23, 24). Specifically, patients with lower SBP (<120 mmHg) at admission had higher in-hospital and post-discharge mortality rates, while higher SBP at admission was associated with lower in-hospital mortality rates. Moreover, Shiraishi et al. examined the prognostic impact of mean arterial pressure (MAP) on acute MI patients' in-hospital outcomes. Their results showed that patients with lower admission MAP (<79 mmHg) presented worse in-hospital prognosis (15). Interestingly, obesity (BMI ≥ 25 kg/m^2^) was significantly related to higher average SBP, DBP, and MAP (16, 25, 26). These observations indicated an inhibitive role of obesity in the progression of MI. Our results partially explain the lower mortality rates of acute MI patients with obesity (6, 7).

Results of mega-analysis indicated that PTH could be a novel gene involved in the pathogenesis of MI. This gene (PTH) demonstrated significantly lower expression in MI patients (LFC = −45.97; *p*-value < 1E-324). Nevertheless, our literature-based network analysis suggested that PTH could play a mixed role in the pathogenesis of MI. For instance, PTH administration could stimulate the expression of MMP9, which plays an important role in the onset and prognosis of MI (27, 28). However, PTH has also been shown to increase ß-catenin expression and Wnt/ß-MI development (29–32). Besides, PTH expression has demonstrated significant variance among MI patients, which was influenced by sample size and study age (*p* < 1e^−324^). Therefore, the relationship between PTH and MI needs further validation.

## Conclusions

Our study suggested that obesity could influence MI progression by driving multiple genes associated with blood pressure regulation. Moreover, PTH could be a novel obesity driven gene associated with MI, which needs further validation.

## Data Availability Statement

The original contributions presented in the study are included in the article/Supplementary Material, further inquiries can be directed to the corresponding author.

## Author Contributions

SYZ designed the study, performed computational analysis, and drafted the manuscript. RC, SHZ, and YK contributed to the data analysis and manuscript writing. All authors read and approved the final version of the manuscript.

## Conflict of Interest

The authors declare that the research was conducted in the absence of any commercial or financial relationships that could be construed as a potential conflict of interest.

## References

[1] Zhu J Su X Li G Chen J Tang B Yang Y. The incidence of acute myocardial infarction in relation to overweight and obesity: a meta-analysis. Arch Med Sci. (2014) 10:855–62. 10.5114/aoms.2014.46206 25395935 PMC4223131

[2] Yusuf S Hawken S Ounpuu S Bautista L Franzosi MG Commerford P. Obesity and risk of myocardial infarction in 27,00 participants from 52 countries: a case–control study. Lancet. (2005) 366:1640–9. 10.1016/S0140-6736(05)67663-5 16271645

[3] Schargrodsky H Rozlosnik J Ciruzzi M Ruffa R Paterno C Ardariz M. Body weight and nonfatal myocardial infarction in a case-control study from Argentina. Soz Praventivmed. (1994) 39:126–33. 10.1007/BF01299656 8048272

[4] Yusuf S Hawken S Ounpuu S Dans T Avezum A Lanas F. Effect of potentially modifiable risk factors associated with myocardial infarction in 52 countries (the INTERHEART study): case-control study. Lancet. (2004) 364:937–52. 10.1016/S0140-6736(04)17018-9 15364185

[5] Xu Y Li H Wang A Su Z Yang G Luo Y. Association between the metabolically healthy obese phenotype and the risk of myocardial infarction: results from the Kailuan study. Eur J Endocrinol. (2018) 179:343–52. 10.1530/EJE-18-0356 30400019

[6] Mehta L Devlin W McCullough PA O'Neill WW Skelding KA Stone GW. Impact of body mass index on outcomes after percutaneous coronary intervention in patients with acute myocardial infarction. Am J Cardiol. (2007) 99:906–10. 10.1016/j.amjcard.2006.11.038 17398181

[7] Dhoot J Tariq S Erande A Amin A Patel P Malik S. Effect of morbid obesity on in-hospital mortality and coronary rrevascularization outcomes after acute myocardial infarction in the United States. Am J Cardiol. (2013) 111:1104–10. 10.1016/j.amjcard.2012.12.033 23360768 PMC3885329

[8] Krzemiński TF Nozyński JK Grzyb J Porc M Zegleń S Filas V. Angiogenesis and cardioprotection after TNFalpha-inducer-Tolpa Peat Preparation treatment in rat's hearts after experimental myocardial infarction in vivo. Vascul Pharmacol. (2005) 43:164–70. 10.1016/j.vph.2005.06.003 16043419

[9] Dogusal G Afacan B Bozkurt E Sönmez I. Gingival crevicular fluid and salivary resistin and tumor necrosis factor-alpha levels in obese children with gingivitis. J Periodontol. (2018) 89:973–82. 10.1002/JPER.17-0613 29635809

[10] Tian M Yuan Y Li J Gionfriddo M Huang R. Tumor necrosis factor-α and its role as a mediator in myocardial infarction: a brief review. Chronic Dis Transl Med. (2015) 1:18–26. 10.1016/j.cdtm.2015.02.002 29062983 PMC5643772

[11] Celik Guzel E Bakkal E Guzel S Eroglu HE Acar A Kuçukyalcin V. Can low brain-derived neurotrophic factor levels be a marker of the presence of depression in obese women? Neuropsychiatr Dis Treat. (2014) 10:2079–86. 10.2147/NDT.S72087 25395856 PMC4226451

[12] Halade GV Ma Y Ramirez TA Zhang J Dai Q Hensler JG. Reduced BDNF attenuates inflammation and angiogenesis to improve survival and cardiac function following myocardial infarction in mice. Am J Physiol Heart Circ Physiol. (2013) 305:H1830–H1842. 10.1152/ajpheart.00224.2013 24142413 PMC3882541

[13] Borenstein M Hedges LV Higgins JP Rothstein HR. A basic introduction to fixed-effect and random-effects models for meta-analysis. Res Synth Methods. (2010) 1:97–111. 10.1002/jrsm.12 26061376

[14] Vesztrocy AW Dessimoz C. A gene ontology tutorial in python. Methods Mol Biol. (2017) 1446:221–9. 10.1007/978-1-4939-3743-1_16 27812946

[15] Shiraishi J Nakamura T Shikuma A Shoji K Nishikawa M Yanagiuchi T. Relationship Between Mean Blood Pressure at Admission and In-Hospital Outcome After Primary Percutaneous Coronary Intervention for Acute Myocardial Infarction. Int Heart J. (2016) 57:547–52. 10.1536/ihj.15-480 27535713

[16] Toyoshima H Otsuka R Hashimoto S Tamakoshi K Yatsuya H. Body mass index-modified relationship of chronic mental stress with resting blood pressure during 5 years in Japanese middle-aged male workers. Circ J. (2014) 78:1379–86. 10.1253/circj.CJ-13-1086 24705468

[17] Nakagawa K Umetani K Fujioka D Sano K Nakamura T Kodama Y. Correlation of plasma concentrations of B-type natriuretic peptide with infarct size quantified by tomographic thallium-201 myocardial scintigraphy in asymptomatic patients with previous myocardial infarction. Circ J. (2004) 68:923–7. 10.1253/circj.68.923 15459465

[18] Yamamoto A Tanabe K Yokoyama Y Itoh H Murayama M. Influence of aerobic exercise training on brain natriuretic peptide secretion in patients in the chronic phase of myocardial infarction. Jpn Circ J. (1998) 62:658–64. 10.1253/jcj.62.658 9766703

[19] Fukuta H Ohte N Wakami K Goto T Tani T Kimura G. Decreased plasma B-type natriuretic peptide levels in obesity are not explained by altered left ventricular hemodynamics. Obes Res Clin Pract. (2011) 5:e267–360. 10.1016/j.orcp.2011.04.001 24331139

[20] Zhang J Li XX Bian HJ Liu XB Ji XP Zhang Y. Inhibition of the activity of Rho-kinase reduces cardiomyocyte apoptosis in heart ischemia/reperfusion via suppressing JNK-mediated AIF translocation. Clin Chim Acta. (2009) 401:76–80. 10.1016/j.cca.2008.11.016 19061880

[21] Kitano K Usui S Ootsuji H Takashima S Kobayashi D Murai H. Rho-kinase activation in leukocytes plays a pivotal role in myocardial ischemia/reperfusion injury. PLoS ONE. (2014) 9:e92242. 10.1371/journal.pone.0092242 24638037 PMC3956925

[22] O'Brien M Carbin S Morrison JJ Smith TJ. Decreased myometrial p160 ROCK-1 expression in obese women at term pregnancy. Reprod Biol Endocrinol. (2013) 11:79. 10.1186/1477-7827-11-79 23948067 PMC3765888

[23] Gheorghiade M Abraham WT Albert NM Greenberg BH O'Connor CM She L. Systolic blood pressure at admission, clinical characteristics, and outcomes in patients hospitalized with acute heart failure. JAMA. (2006) 296:2217–26. 10.1001/jama.296.18.2217 17090768

[24] Shiraishi J Kohno Y Sawada T Hashimoto S Ito D Kimura M. Prognostic impact of systolic blood pressure at admission on in-hospital outcome after primary percutaneous coronary intervention for acute myocardial infarction. J Cardiol. (2012) 60:139–44. 10.1016/j.jjcc.2012.02.008 22521431

[25] Guedes-Martins L Carvalho M Silva C Cunha A Saraiva J Macedo F. Relationship between body mass index and mean arterial pressure in normotensive and chronic hypertensive pregnant women: a prospective, longitudinal study. BMC Pregnancy Childbirth. (2015) 15:281. 10.1186/s12884-015-0711-0 26518235 PMC4628392

[26] Tang B Luo F Zhao J Ma J Tan I Butlin M. Relationship between body mass index and arterial stiffness in a health assessment Chinese population. Medicine. (2020) 99:e18793. 10.1097/MD.0000000000018793 32011479 PMC7220472

[27] Squires CE Escobar GP Payne JF Leonardi RA Goshorn DK Sheats NJ. Altered fibroblast function following myocardial infarction. J Mol Cell Cardiol. (2005) 39:699–707. 10.1016/j.yjmcc.2005.07.008 16111700

[28] Mukherjee R Brinsa TA Dowdy KB Scott AA Baskin JM Deschamps AM. Myocardial infarct expansion and matrix metalloproteinase inhibition. Circulation. (2003) 107:618–25. 10.1161/01.CIR.0000046449.36178.00 12566376

[29] Fu WB Wang WE Zeng CY. Wnt signaling pathways in myocardial infarction and the therapeutic effects of Wnt pathway inhibitors. Acta Pharmacol Sin. (2019) 40:9–12. 10.1038/s41401-018-0060-4 30002488 PMC6318317

[30] Templin C Kotlarz D Faulhaber J Schnabel S Grote K Salguero G. Ex vivo expanded hematopoietic progenitor cells improve cardiac function after myocardial infarction: role of beta-catenin transduction and cell dose. J Mol Cell Cardiol. (2008) 45:394–403. 10.1016/j.yjmcc.2008.06.010 18671980

[31] Dawson K Aflaki M Nattel S. Role of the Wnt-Frizzled system in cardiac pathophysiology: a rapidly developing, poorly understood area with enormous potential. J Physiol. (2013) 591:1409–32. 10.1113/jphysiol.2012.235382 23207593 PMC3607163

[32] Hahn JY Cho HJ Bae JW Yuk HS Kim KI Park KW. Beta-catenin overexpression reduces myocardial infarct size through differential effects on cardiomyocytes and cardiac fibroblasts. J Biol Chem. (2006) 28:30979–89. 10.1074/jbc.M603916200 16920707

